# XB130 promotes proliferation and invasion of gastric cancer cells

**DOI:** 10.1186/1479-5876-12-1

**Published:** 2014-01-04

**Authors:** Min Shi, Dayong Zheng, Li Sun, Lin Wang, Li Lin, Yajun Wu, Minyu Zhou, Wenjun Liao, Yulin Liao, Qiang Zuo, Wangjun Liao

**Affiliations:** 1 Department of Oncology, Nanfang Hospital, Southern Medical University, 1838 North Guangzhou Avenue, 510515 Guangzhou, China; 2 Department of Cardiology, Nanfang Hospital, Southern Medical University, 510515 Guangzhou, China

**Keywords:** Gastric cancer, Adaptor protein, Oncogene, Epithelial-mesenchymal transition-like

## Abstract

**Background:**

XB130 has been reported to be expressed by various types of cells such as thyroid cancer and esophageal cancer cells, and it promotes the proliferation and invasion of thyroid cancer cells. Our previous study demonstrated that XB130 is also expressed in gastric cancer (GC), and that its expression is associated with the prognosis, but the role of XB130 in GC has not been well characterized.

**Methods:**

In this study, we investigated the influence of XB130 on gastric tumorigenesis and metastasis in vivo and in vitro using the MTT assay, clonogenic assay, BrdU incorporation assay, 3D culture, immunohistochemistry and immunofluorescence. Western blot analysis was also performed to identify the potential mechanisms involved.

**Results:**

The proliferation, migration, and invasion of SGC7901 and MNK45 gastric adenocarcinoma cell lines were all significantly inhibited by knockdown of XB130 using small hairpin RNA. In a xenograft model, tumor growth was markedly inhibited after shXB130-transfected GC cells were implanted into nude mice. After XB130 knockdown, GC cells showed a more epithelial-like phenotype, suggesting an inhibition of the epithelial-mesenchymal transition (EMT) process. In addition, silencing of XB130 reduced the expression of p-Akt/Akt, upregulated expression of epithelial markers including E-cadherin, α-catenin and β-catenin, and downregulated mesenchymal markers including fibronectin and vimentin. Expression of oncoproteins related to tumor metastasis, such as MMP2, MMP9, and CD44, was also significantly reduced.

**Conclusions:**

These findings indicate that XB130 enhances cell motility and invasiveness by modulating the EMT-like process, while silencing XB130 in GC suppresses tumorigenesis and metastasis, suggesting that it may be a potential therapeutic target.

## Background

XB130 is a newly identified adaptor protein that is expressed in the spleen, thyroid, and esophagus in humans [1,2]. It has also been detected in follicular and papillary thyroid carcinoma cell lines [3]. As a tumor promoter, XB130 has been found to enhance cell proliferation, metastasis, and resistance to cell death, as well as being involved in signal transduction in thyroid cancer cells [3]. Our previous study revealed that XB130 is expressed in gastric cancer (GC) and that its expression can predict the survival prognosis and chemotherapeutic-sensitivity [4], suggesting that XB130 plays an important role in GC. However, the detailed mechanisms by which XB130 acts in GC remain poorly defined.

As a member of the actin filament-associated protein (AFAP) family of adaptor proteins, XB130 has been reported to display a high affinity for lamellipodial (branched) F-actin and to influence thyroid cancer cell motility and invasiveness [5]. Lamellipodia are essential for the formation of migratory membrane protrusions, an event that is closely related to the epithelial-mesenchymal transition (EMT). The EMT is the process by which epithelial cells undergo a phenotypic change to become mesenchymal cells and it is a key step in tumor invasion and metastasis [6]. Several signaling pathways are involved in this process, including those mediated by focal adhesion kinase (FAK)/Src, phosphatidyl inositol 3-kinase (PI3K)/Akt, and mitogen-activated protein kinase (MAPK) [7-9]. It has been showed that XB130 is involved in the activation of Akt [10,11], while Xu et al. demonstrated that XB130 participates in activation of the c-Src pathway [1]. Intriguingly, these signaling pathways have been reported to play an essential role in the development and progression of GC [12-14], suggesting that XB130 could also be a pro-metastatic factor for GC. However, whether XB130 is involved in promoting the EMT process and metastasis of GC remains undetermined.

In the present study, we used XB130-silenced cell lines that we established in previous study [4] to investigate the influence of XB130 on GC both in vitro and in vivo. Our hypothesis was that XB130 would promote GC proliferation and invasion, as well as having a role in the EMT.

## Methods

### Cell lines and reagents

Several common human gastric adenocarcinoma cell lines [(BGC823), (SGC7901), (MKN45), (MKN28), (AGS)] were obtained from Foleibao Biotechnology Development Company (Shanghai, China). Cells were cultured in complete medium [Roswell Park Memorial Institute 1640 medium (Life Technologies, Carlsbad, CA, USA) with 10% fetal bovine serum (Thermo Scientific HyClone, South Logan, UT, USA)] at 37°C under 5% CO_2_. Cells were harvested in the logarithmic growth phase for use in the experiments described below. Silencing of XB130 was carried out using small hairpin RNA (shRNA) as described previously [4]. The sequences were GCTGAAGATCACACCGATG for XB130-silencing shRNA and GCCAGCTTAGCACTGACTC for Scramble shRNA, respectively. Establishment of cell lines transfected with XB130 shRNA (sh-XB130) was performed as described previously [4].

Rabbit antibodies for fibronectin and CD44, as well as mouse antibodies for E-cadherin, vimentin, α-catenin, β-catenin, XB130 and β-actin were purchased from Santa Cruz Biotechnology Company (Santa Cruz, CA, USA). Rabbit antibodies for β-actin, Akt, and p-Akt were purchased from Cell Signaling Technology Company (Boston, MA, USA), while a rabbit antibody targeting XB130 was obtained from PradoWalnut Company (Walnut, CA, USA).

### Fluorescence quantitative real-time polymerase chain reaction (RT-PCR)

Primer sequences for human XB130 were (F) 5′-AAGCAGCAGCTCTGATGAGG-3′ and (R) 5′-GGTCTGGAAGGCTCTTCTGA-3′. Total RNA was extracted from cultured cells using a Trizol kit (Life Technologies, Carlsbad, CA, USA). Then cDNA was synthesized using total RNA and MMLV-RT reverse transcriptase (ProSpec, East Brunswick, NJ, USA). The reaction mixture for RT-PCR was prepared according to the manufacturer’s protocol.

### Western blotting

Cells were lysed on ice in RIPA buffer [50 mM Tris-Cl (pH 7.5), 120 mM NaCl, 10 mM NaF, 10 mM sodium pyrophosphate, 2 mM EDTA, 1 mM Na_3_VO_4_, 1 mM PMSF, and 1% NP-40] containing a protease inhibitor cocktail (Roche, Basel, CH). The protein content of the lysates was determined by the method of Bradford. Approximately 50–75 μg of protein was resolved by 8% or 12% sodium dodecyl sulfate polyacrylamide gel electrophoresis and was transferred to nitrocellulose membranes (Millipore, Bedford, MA, USA). The membranes were blocked in TBST [25 mM Tris–HCl (pH 7.5), 125 mM NaCl, and 0.1% Tween 20] containing 5% bovine serum albumin (BSA), and then incubated with primary antibodies targeting XB130, E-cadherin, α-catenin, β-catenin, fibronectin, MMP9, MMP2, vimentin, CD44, Akt, p-Akt, or β-actin in TBST containing 1% BSA overnight at 4°C. Subsequently, incubation was done with the appropriate secondary antibodies for 1 h at room temperature. Reactive protein bands were visualized with a Western Lightning Plus-ECL (Perkin Elmer, Waltham, MA, USA) after exposure to radiographic film and were quantified with QuantityOne v4.6.2 imaging software (Bio-Rad, Hercules, CA, USA).

### Clonogenic assay

To investigate the ability of cells to form colonies, 1×10^3^ cells transfected with XB130 shRNA or Scramble RNA were seeded into 6-well plates and incubated for 2 weeks with a medium change every 3–4 days. Colonies were stained with 0.05% crystal violet (Sigma Chemical Company, Louis, MO, USA) for 1 h at room temperature, washed twice with phosphate-buffered saline (PBS), and observed under a microscope (Olympus, Tokyo, Japan).

### Soft agar colony-forming assay

Cells were trypsinized and suspended in 2 mL of complete medium with 0.3% agar (Sigma Chemical Company, Louis, MO, USA), and then the agar-cell mixture was plated onto the bottom layer with 1% agar in complete medium. After being cultured in an incubator for 4 weeks, cells were observed and photographed under a microscope.

### Cell viability assay

After trypsinization, cells were seeded into 96-well plates at a density of 0.2×10^4^/well for culture, and cell proliferation was measured by the methyl thiazolyl tetrazolium (MTT) assay on days 1, 3, 5, and 7. Briefly, 0.02 mL of MTT solution (5 mg/mL in PBS) was added to each well and incubation was performed for 4 h at 37°C, after which the medium was replaced by 0.15 mL of dimethyl sulfoxide and incubation was done for 10 min. Then the optical density was measured at 492 nm with a Microplate spectrophotometer (Thermo Scientific, Pittsburgh, PA, USA).

### Cell cycle analysis

Cell cycle analysis was performed by flow cytometry (Beckman-coulter, Fullerton, CA, USA) after staining the cells with propidium iodide (PI) (Sigma Chemical Company, Louis, MO, USA). Cells were harvested by trypsinization, washed with PBS, and fixed in 70% ethanol for 30 min on ice. Then the cells were washed again, resuspended in PBS containing Triton-X-100 and 2 mg/mL RNase A (Thermo Scientific, Pittsburgh, PA, USA), and incubated at 37°C for 30 min. Next, PI was added at a final concentration of 25 μg/mL and the cells were incubated on ice for 30 min. After staining with PI had been completed, a minimum of 10,000 events were counted for each sample by flow cytometry and the cell cycle profile was analyzed with Flowjo software (TreeStar, Ashland, Oregon, USA).

### BrdU incorporation assay

The effect of XB130 inhibition on DNA synthesis was determined by estimating the uptake of 5-bromo-2′deoxyuridine-5′monophophate (BrdU) into DNA. Cells in the logarithmic growth phase were trypsinized, transferred to a sterile coverslip, and incubated until they became adherent. After serum starvation for 48 h and incubation in complete medium for 4 h, the cells were labeled with 10 μmol/L BrdU for 1 h. Then the cells were fixed and permeablilized with 0.1% Triton and 0.1% citric acid for 10 min at room temperature, after which endogenous peroxidase was blocked by incubation with 3% hydrogen peroxide (H_2_O_2_) for 10 min at room temperature. For nuclear staining, cells were incubated in serum-free medium with anti-BrdU antibody for 1 h at 37°C. Each experiment was repeated 3 times independently, and stained cells were counted under a fluorescence microscope (Olympus, Tokyo, Japan).

### Wound healing assay

SGC7901 and MNK45 cells were seeded into 6-well plates at 90% confluence and incubated overnight for adherence. Then a wound was made along the center of each well by scratching the cell layer with the tip of a 200 μL pipette. Next, the wells were washed twice with PBS to remove loose cells and fresh medium was added. Photographs were taken at 0 h, 10 h, and 24 h to assess cell migration into the wound.

### Transwell invasion assay

The invasive potential of wild-type and XB130-silenced GC cells was assessed by an invasion assay using 24-well Matrigel invasion chambers (BD Biosciences, San Jose, CA, USA). Briefly, Matrigel inserts and an equal number of control inserts were prepared according to the manufacturer’s protocol. SGC7901 cells and MNK45 cells (5×10^4^/mL in 0.5 mL of serum-free medium) were added to the upper chambers, and 0.75 mL of medium supplemented with 5% fetal bovine serum was added to each of the lower chambers as a chemoattractant. After incubation for 22 h, the cells remaining in the upper chambers were removed by scraping, and the invading cells in the lower chambers were fixed with 3.7% paraformaldehyde. Then the cells were washed twice with PBS, stained with hematoxylin for 1 h at room temperature, and photographed under a microscope.

### 3D Culture in matrigel

Twenty-four–well dishes were coated with 100 μL of growth factor reduced solidified Matrigel (BD Biosciences, San Jose, CA, USA) and placed in an incubator. The cells were trypsinized and were seeded at a density of 500 per well in 500 μL of medium. After incubation for 2 weeks, the cultures were photographed under a microscope.

### Immunofluorescence

Cells were grown on coverslips, fixed with 4% paraformaldehyde for 30 min, and washed three times with PBS. Then the cells were permeabilized with 0.2% Triton X-100 for 5 min at room temperature and blocked with 1% BSA for 1 h. Next, incubation was done with primary antibodies targeting XB130, E-cadherin, and vimentin overnight at 4°C, followed by incubation with appropriate secondary antibodies (Alexa Fluor® 594 or Alexa Fluor® 488) for 1 h at room temperature. Nuclei were counterstained with 4′,6-diamidino-2-phenylindole (Life Technologies, Carlsbad, CA, USA), while F-actin filaments were stained with rhodamine phalloidin (Sigma Chemical Company, Louis, MO, USA), and the cells were viewed with a confocal laser-scanning microscope.

### Xenograft model in nude mice

Six-week-old Balb/c nude mice were purchased from Sun Yat-Sen University (Guangzhou, China). All experimental procedures involving animals were done in accordance with the Guide for the Care and Use of Laboratory Animals and conformed to our institutional ethical guidelines for animal experiments. ShXB130-transfected, empty plasmid-transfected, and untransfected SGC7901 cells were trypsinized, collected by centrifugation, and suspended in RPMI-1640 medium. Then 0.2 mL of medium containing 1×10^7^ cells was injected subcutaneously into the left and right posterior flank regions of each mouse. The mice were housed in a pathogen-free environment and tumor growth was monitored every 3 days. Mice were killed after 21 days and the volume of each tumor was calculated according to the formula V = a×b×(a + b)/2, where “a” and “b” are respectively the length and the width of the tumor measured with a sliding caliper.

### Immunohistochemistry

Sections (4 μm) of the xenograft tumor tissues were subjected to immunohistochemical staining as follows. Formalin-fixed and paraffin-embedded tissue sections were deparaffinized in xylene, rehydrated in a graded alcohol series, and washed with PBS. Then the sections were immersed in 10 mmol/L citrate buffer (pH 6.0) and heated in a microwave for 30 min. After cooling to room temperature, endogenous peroxidase was blocked by incubation with 3% H_2_O_2_ in methanol. Nonspecific binding was blocked by incubating the sections with 1% BSA in a humid chamber for 60 min. Incubation with the primary antibodies was subsequently performed overnight at 4°C using antibodies for XB130, E-cadherin, vimentin, or p-Akt. Then incubation with suitable secondary antibodies was done in PBS with 0.3% Triton X-100/5% horse serum albumin for 1 h in a humidified chamber. Detection was performed with a Dako Envision System (Dako, Glostrup, Denmark) after slides were counterstained with hematoxylin. Isotype-matched IgG (at the same dilution as the primary antibodies) was used as the negative control.

### Statistical analysis

SPSS 13.0 software was employed for statistical analysis. Results are reported as the mean±SEM. One-way ANOVA was done with Bonferroni’s multiple comparison exact probability test, and Student’s t-test was used to compare continuous variables between two groups. Statistical significance was accepted at *p* < 0.05.

## Results

### Silencing XB130 inhibits proliferation of GC cell lines

**Figure 1:**
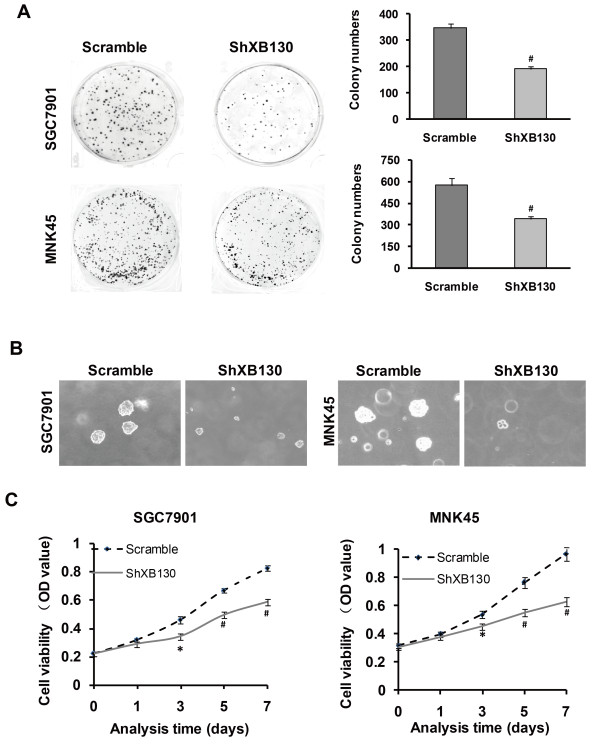
**Silencing of XB130 inhibited proliferation of both SGC7901 and MNK45 gastric cancer cells. (A)** In the plate colony forming assay, fewer colonies were formed by sh-XB130-transfected (sh-XB130) cells than by scramble shRNA-transfected (Scramble) cells. **(B)** In soft agar culture, the number of colonies was significantly smaller for sh-XB130 cells (^#^*p* < 0.01 versus Scramble cells, n = 3 plates per group). Magnification: ×200. **(C)** In the MTT assay, viability of sh-XB130 cells was markedly suppressed in a time-dependent manner. *p < 0.05 and ^#^*p* < 0.01 versus the corresponding time for Scramble cells, n = 3 (6 holes for each time point in each group).

**Figure 2:**
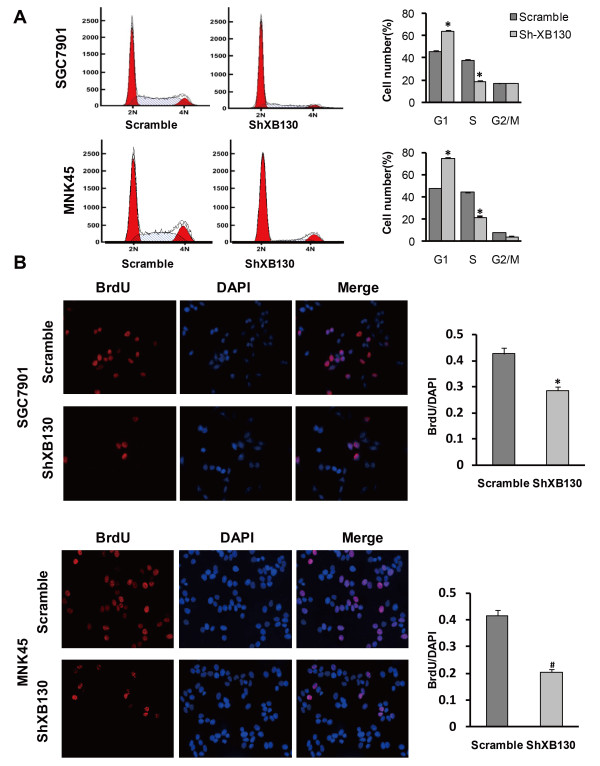
**XB130 knockdown inhibited cell cycle progression and DNA synthesis by GC cells. (A)** XB130 silencing arrested cell cycle progression in both cell lines, as indicated by accumulation of cells in G1 phase and reduced progression into S phase. Cell cycle analysis was performed by flow cytometry using propidium iodide staining. (^#^*p* < 0.01 versus Scramble cells, n = 3). **(B)** The BrdU uptake assay showed a decrease of DNA synthesis in sh-XB130 transfected cells (newly synthesized DNA is stained red by BrdU, while nuclei are stained blue by 4′, 6-diamidino-2-phenylindole). *p < 0.05 and ^#^*p* < 0.01 versus Scramble cells (n = 3 per group).

Among the 5 common human GC cell lines, we found that XB130 expression was higher in SGC7901 (a poorly-differentiated cell line) and MKN45 (a well-differentiated cell line) than in the other cell lines (Additional file 1: Figure S1A). Accordingly, we chose these two cell lines for transfection with sh-XB130. The knockdown effect of sh-XB130 was confirmed by real-time PCR (Additional file 1: Figure S1B) and Western blotting (Additional file 1: Figure S1C). Compared with Scramble shRNA-transfected cells (Scramble cells), colony formation by sh-XB130-transfected cells (sh-XB130 cells) was markedly reduced in the plate colony forming assay (Figure 1A). In addition, the number of colonies that grew in soft agar was significantly reduced by transfection of sh-XB130 (*p* < 0.01) (Figure 1B). When the MTT assay was used to assess cell viability over a period of 7 days, we found that viability was significantly lower in sh-XB130 cells than in Scramble cells, indicating that cell viability was suppressed by knockdown of XB130 (Figure 1C). Cell cycle analysis revealed that sh-XB130 cells were arrested in G1 phase, accompanied by a significant reduction of cells in S phase (Figure 2A). The BrdU labeling assay showed that DNA synthesis was also strongly inhibited in sh-XB130 cells (Figure 2B). These results indicate that cell proliferation was remarkably inhibited by silencing of XB130.

### Silencing XB130 inhibits GC cell motility and invasiveness and alters the phenotype of GC cells

**Figure 3:**
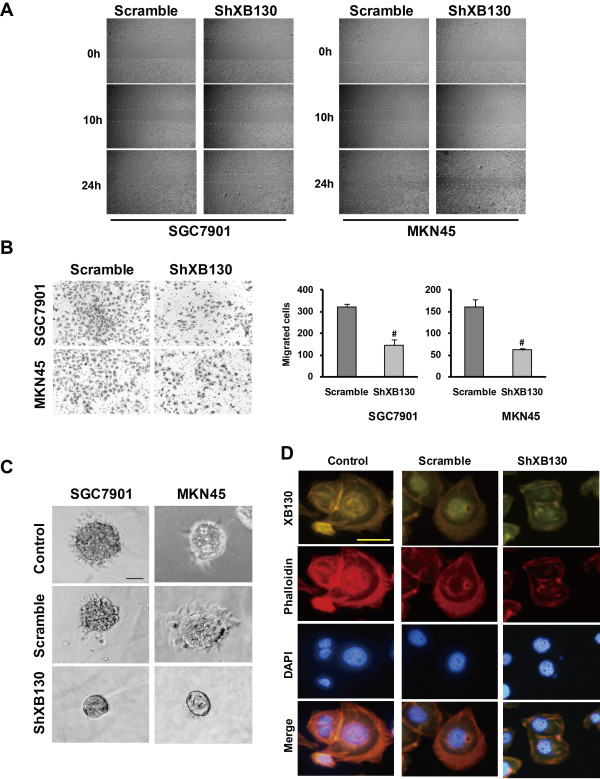
**Silencing of XB130 inhibited migration and invasion of SGC7901 cells and MNK45 cells, and inhibited the phentotypic transition. (A)** Cell migration was assessed by the wound healing assay. The residual wound was much wider in the sh-XB130 group than in the Scramble group at 24 hours. **(B)** In the Transwell migration assay, the number of cells migrating into the lower chamber was much smaller in the sh-XB130 group than in the Scramble group. (^#^*p* < 0.01 versus the Scramble group, n = 3). **(C)** Matrigel 3D culture was performed to assess cell morphology. Aggressive protrusions were seen in both the Control group and the Scramble group. In contrast, the invasiveness of sh-XB130 transfected cells was much lower and the cells were round spheroids with few or no protrusions. Scale bar = 100 μm. Similar results were obtained with both SGC7901 cells and MNK45 cells. **(D)** SGC7901 cells were immunostained with an XB130 antibody and F-actin filaments were counterstained using rhodamine phalloidin, while the nuclei were stained with 4′,6-diamidino-2-phenylindole. All experiments were repeated 3 times.

To assess the effect of down-regulation of XB130 on cell motility, the wound healing assay and Transwell assay were performed. After knockdown of XB130, we found that fewer cells migrated to the center of the wound in the wound healing assay (Figure 3A) or migrated into the lower chamber in the Transwell assay (*p* < 0.01) (Figure 3B). In addition, sh-XB130 cells were relatively smooth spheroids with few projections, while Scramble cells and Control cells developed a multipolar invasive morphology in 3D culture (Figure 3C and Additional file 2: Figure S2A). We also investigated the cell structure by staining F-actin filaments. We found that XB130 was expressed in the F-actin filaments and XB130 knockdown resulted in GC cells adopting an epithelial-like morphology (Figure 3D). These findings indicate that the motility of GC cells was suppressed along with a decrease of invasive morphologic features after down-regulation of XB130.

### Silencing XB130 reduces tumor growth in nude mice

**Figure 4:**
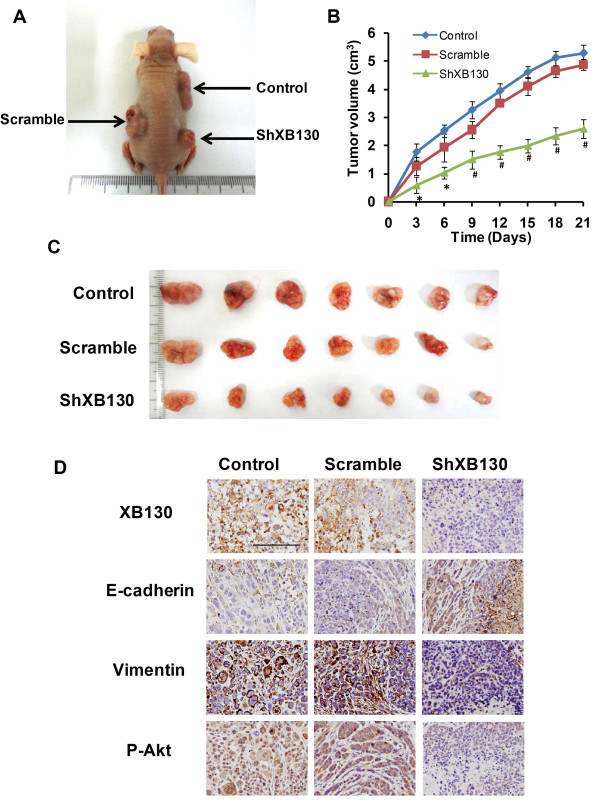
**XB130 silencing reduced the growth of xenograft tumors.** SGC7901 cells were implanted in nude mice by subcutaneous injection into the anterior right flank, while the same amounts of wild-type, Scramble RNA-transfected, or sh-XB130-transfected SGC7901 cells were injected into the posterior left and right flanks. **(A)** After subcutaneous injection, tumors always formed, as shown in this representative picture taken on day 21. **(B)** Growth curves of the tumors. Tumor volumes were measured over 21 days in each group. n = 5 per group, *p < 0.05 and ^#^*p* < 0.01. **(C)** Tumors harvested from the three groups on day 21. **(D)** Representative images of immunostaining for XB130, E-cadherin, vimentin, and p-Akt in xenograft tumors. Scale bar = 100 μm. n = 3 in each group.

To determine the influence of XB130 on tumor growth in vivo, a xenograft nude mouse model was used. Colonies of sh-XB130 cells, Scamble shRNA cells, and Control SGC7901 cells were prepared. After subcutaneous injection into nude mice, all three types of cells formed tumors (Figure 4A). However, tumor growth was much slower after injection of sh-XB130 cells than after injection of Control or Scramble cells (Figure 4B). After 3 weeks, tumor volume was significantly smaller in the sh-XB130 group than in the Control and Scramble groups (*p <* 0.01, Figure 4B and C). These findings indicate that GC tumor growth was inhibited by downregulation of XB130.

### XB130 activates the PI3K/Akt pathway and alters both EMT markers and metastasis-associated proteins in GC

**Figure 5:**
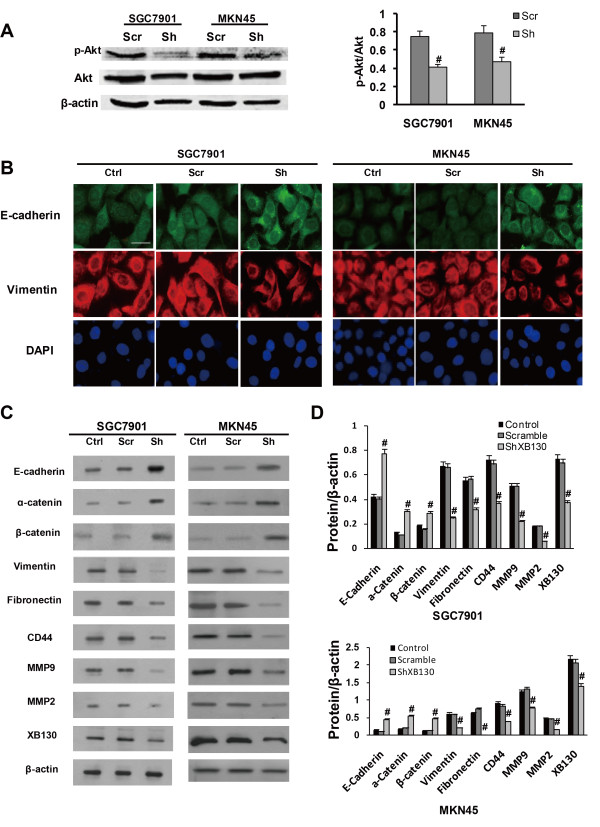
**Silencing of XB130 modulated phosphorylation of Akt and expression of EMT-associated proteins by cultured GC cell lines. (A)** Phosphorylation of Akt was significantly decreased by XB130 knockdown. **(B)** Immunostaining for E-cadherin and vimentin. E-cadherin was significantly upregulated and vimentin was downregulated by XB130 knockdown. Scale bar = 50 μm. n = 3 in each group. **(C)** Representative results of Western blotting. In comparison with the Scramble group, silencing of XB130 upregulated the expression of E-cadherin, α-catenin and β-catenin, while it downregulated expression of fibronectin, vimentin, CD44, MMP9 and MMP2. **(D)** Quantitative analysis of panel C. ^#^*p* < 0.01 versus the Scramble group, n = 3. Ctrl = control, Scr = Scramble, Sh = sh-XB130.

To explore the mechanisms underlying the above-mentioned changes induced by silencing of XB130, we postulated that its downregulation might influence the expression of EMT markers and metastasis-associated proteins via the PI3K/Akt pathway. We found that knockdown of XB130 decreased the phosphorylation of Akt in xenograft GC tissues (Figure 4D) and in GC cell lines (Figure 5A). Immunofluorescence, immunohistochemistry, and Western blotting were combined to assess the expression of EMT markers. In contrast to the Scramble group, silencing of XB130 in xenograft GC tissues and cultured GC cell lines led to higher expression of the epithelial marker E-cadherin and lower expression of the mesenchymal marker vimentin (Figure 4D, Figure 5B, and Additional file 2: Figure S2B). Western blotting also showed that silencing of XB130 substantially increased the expression of epithelial markers (E-cadherin, α-catenin and β-catenin), while causing a significant decrease in the expression of mesenchymal markers (fibronectin and vimentin) and metastasis-associated proteins (MMP2, MMP9 and CD44) (Figure 5C and D).

## Discussion

The present study has provided the first evidence concerning the role of XB130 in GC, showing that (1) XB130 contributes to GC cell proliferation and invasiveness, (2) XB130 is involved in phosphorylation of Akt and EMT-like changes, and (3) XB130 could be a potential therapeutic target in patients with GC.

XB130 was initially cloned as a homologue of actin filament-associated protein (AFAP-110) [1], which has been suggested to have a role in mechanotransduction, stress fiber stabilization, focal adhesion formation, and podosome dynamics [15-17]. Regarding the functional properties of XB130, similar to AFAP-110, it is associated with regulation of the cytoskeleton, exhibits a high affinity for lamellipodial F-actin, and influences the motility and invasiveness of thyroid tumor cells [5]. Consequently, knockdown of XB130 in thyroid cancer cells decreases the wound closure rate, inhibits cell invasion, reduces lamellipodial persistence, and slows cell spreading [5]. Consistent with that report, we found that silencing of XB130 decreased the motility of GC cells along with significant inhibition of the transition from epithelial-like to fibroblast-like morphology, indicating that XB130 affects the motility and invasiveness of these tumor cells by interfering with an EMT-like process.

The EMT is a highly conserved process that has been well characterized in embryogenesis. In epithelial tumors, epithelial-like cancer cells undergo a phenotypic change to become mesenchymal-like cells which is similar to fibroblasts [18,19]. These changes lead to loss of polarity for epithelial cells and resulted in promotion of tumor cell metastasis [6]. However, tumor cells seldom exhibit a complete change from an epithelial to mesenchymal phenotype (as occurs in the embryological setting), but rather show more plastic and dynamic changes that are better classified as “EMT-like” or as a partial EMT [20,21]. Such EMT-like changes have been reported to be important in the metastasis of epithelial tumors [22].

In most cases, downregulation of E-cadherin seems to be the final common pathway of the EMT. Epithelial cells undergoing the EMT tend to develop a spindle-shaped or fibroblast-like morphology, and display increased or new expression of mesenchymal markers, including vimentin and fibronectin [23]. E-cadherin is a cell adhesion molecule that is anchored to the actin cytoskeleton via a complex consisting of α-catenin and β-catenin [24], and it is thought to be the key molecule in the establishment of cell-cell adhesion at adherens junctions. Fibronectin and vimentin are generally considered to be typical mesenchymal markers have been reported to contribute to invasion and distant metastasis of GC [25-27]. In the present study, expression of E-cadherin was significantly increased by XB130 knockdown in vivo and in vitro, while vimentin expression was partially inhibited, suggesting that XB130 has a role in enhancing EMT-like changes of GC.

The PI3K/Akt signaling pathway has been reported to be influenced by XB130 [10,11], and phosphorylation of Akt promotes EMT-like changes through repression of Snail-mediated cadherin 1[7]. MMP2 and MMP9 are members of the matrix metalloproteinase family, which bind to zinc and act on the extracellular matrix (ECM) to degrade type IV collagen in the basement membrane. After basement membrane integrity is lost, metastasis occurs and the survival rate decreases dramatically in GC patients [28-30]. CD44 is recognized as a marker of cancer stem cells, which are a small population of stem-like cells residing in tumor tissues that can cause tumor formation, recurrence, and metastasis [31]. As a transmembrane glycoprotein expressed on the cell surface, CD44 and its variants can bind to the ECM and are involved in making connections between cells and the matrix [31]. All of these extracellular factors contribute to EMT-like changes in tumor cells. In the present study, we found that phosphorylation of Akt, expression of matrix metalloptoteinases, and expression of cancer stem cell markers were all significantly suppressed by XB130 knockdown, further confirming that XB130 may enhance the EMT-like process and promote the motility and invasiveness of GC cells.

As an adaptor protein, XB130 promoted GC cell proliferation and migration, while knockdown of XB130 contributed to reduced growth of xenograft tumors, suggesting that XB130 is an oncoprotein in GC. It may seem paradoxical that our previous study demonstrated a positive correlation between expression of XB130 and the prognosis [4]. In fact, such discrepancy is not uncommon for oncogenes. Several oncogenes are known to be downregulated in tumors and their low expression predicts a poor prognosis. Clinical studies have shown that low expression of the oncoproteins Bcl-2 and Bcl-B is associated with a poor outcome of GC [32-34]. A similar “discrepancy” has also been noted for some tumor suppressor genes. For example, it has been reported that overexpression of the tumor suppressor gene p53 is significantly correlated with unfavorable clinicopathologic parameters and lower overall survival [35]. Moreover, a correlation between gene expression and the prognosis is not necessarily indicative of a causal relationship. Compensatory mechanisms may downregulate some oncogenes and upregulate some tumor suppressor genes.

In addition, clinical prognosis is influenced by various factors including gene expression and medical interventions. Currently, fluoropyrimidine derivative-based and platinum-based combination regimens are accepted as conventional first-line treatment for GC [36]. In our previous study [4], 80% of patients were treated with 5-fluorouracil (5-FU), and XB130-negative patients had a lower survival rate when they received 5-FU. In addition, sensitivity studies showed that XB130 knockdown reduces the sensitivity of GC cells to 5-FU [4]. These results indicate that tumors with high levels of XB130 expression show greater sensitivity to 5-FU, leading to an improved survival rate, which may be an explanation for the better prognosis of patients with high expression of XB130 [4]. Taken together, these findings suggest that XB130 may be a potential target for the treatment of GC.

## Conclusions

In summary, the present study showed that XB130 is an oncogene that promotes tumor growth and metastasis, probably through its role in an EMT-like process. XB130 seems to be an important regulator of the metastasis of gastric cancer and the potential target for treatment of this cancer.

## Abbreviations

GC: Gastric cancer; EMT: Epithelial-mesenchymal transition; PI3K: Phosphatidylinositol 3-kinase; FAK: Focal adhesion kinase; shRNA: Short hairpin RNA; MMP: Matrix metalloproteinase; ECM: Extracellular matrix; 5-FU: 5- fluorouracil; MTT: Methyl thiazolyl tetrazolium; PBS: Phosphate-buffered saline; PI: Propidium iodide.

## Competing interests

The authors declare that they have no competing interests.

## Authors’ contributions

WL, QZ, and MS contributed to conception and design of the study and revised the manuscript. LS, MZ, and WL performed experiments and were responsible for data collection, analysis, and interpretation of the results. LW, LL, and YW were responsible for conducting the data analysis. LS and WL drafted the manuscript and figures, and YL interpreted the data and critically revised the manuscript. All authors read and approved the final manuscript.

## Supplementary Material

Additional file 1: Figure S1 Baseline expression and silencing effect of XB130 in gastric cancer (GC) cell lines. **(A)** Baseline expression of XB130 in various GC cell lines. Knocking down effect of sh-XB130 in SGC7901 and MKN45 cell lines was evaluated. Real-time PCR **(B)** and Western blot **(C)** revealed that sh-XB130 effectively suppressed the expression of XB130 in both cell lines. Click here for file

Additional file 2: Figure S2 Silencing XB130 changed the morphology of gastric cancer cells and altered the EMT-like associated proteins by cultured cells. **(A)** Matrigel 3D culture was performed to assess cell morphology. Aggressive protrusion-positive structures were found in the Scramble and Control group. In contrast, the invasiveness of sh-XB130 transfected cells was much lower and the cells were round spheroids with no or few protrusions. Pictures in upper line showed the view of 2–3 cells in each group under 100× magnification and the others were graphed under 200× magnification. (500 cells were incubated in each well). **(B)** E-cadherin and vimentin immunofluorescence staining. E-cadherin was significantly upregulated and vimentin was downregulated by XB130 knockdown. Scale bar = 100 μm. Experiments were repeated 3 times. Click here for file

## Agents and companies

Cells: Foleibao Biotechnology Development Company, Shanghai, China

Fetal bovine serum: Thermo Scientific HyClone, South Logan, UT, USA

Trizol kit, 4′,6-diamidino-2-phenylindole, and RPMI-1640 medium: Life Technologies, Carlsbad, CA, USA

Protease inhibitor cocktail: Roche, Basel, CH

MMLV-RT reverse transcriptase: ProSpec, East Brunswick, NJ, USA

Nitrocellulose membranes: Millipore, Bedford, MA, USA

Western Lightning Plus-ECL: Perkin Elmer, Waltham, MA, USA

Crystal violet, agar, and propidium iodide: Sigma Chemical Company, Louis, MO, USA

Microplate spectrophotometer and RNase A: Thermo Scientific, Pittsburgh, PA, USA

Flow cytometry: Beckman-coulter, Fullerton, CA, USA

Flowjo software: TreeStar, Ashland, Oregon, USA

Microscope: Olympus, Tokyo, Japan

Matrigel: BD Biosciences, San Jose, CA, USA

Nude mice: Sun Yat-Sen University, Guangzhou, China

Dako Envision System: Dako, Glostrup, Denmark
