# Supplementary figures and images for: XB130 promotes proliferation and invasion of gastric cancer cells
Source: J Transl Med. 2014 Jan 4;12:1. doi: 10.1186/1479-5876-12-1 (PMC3882781; doi:10.1186/1479-5876-12-1)

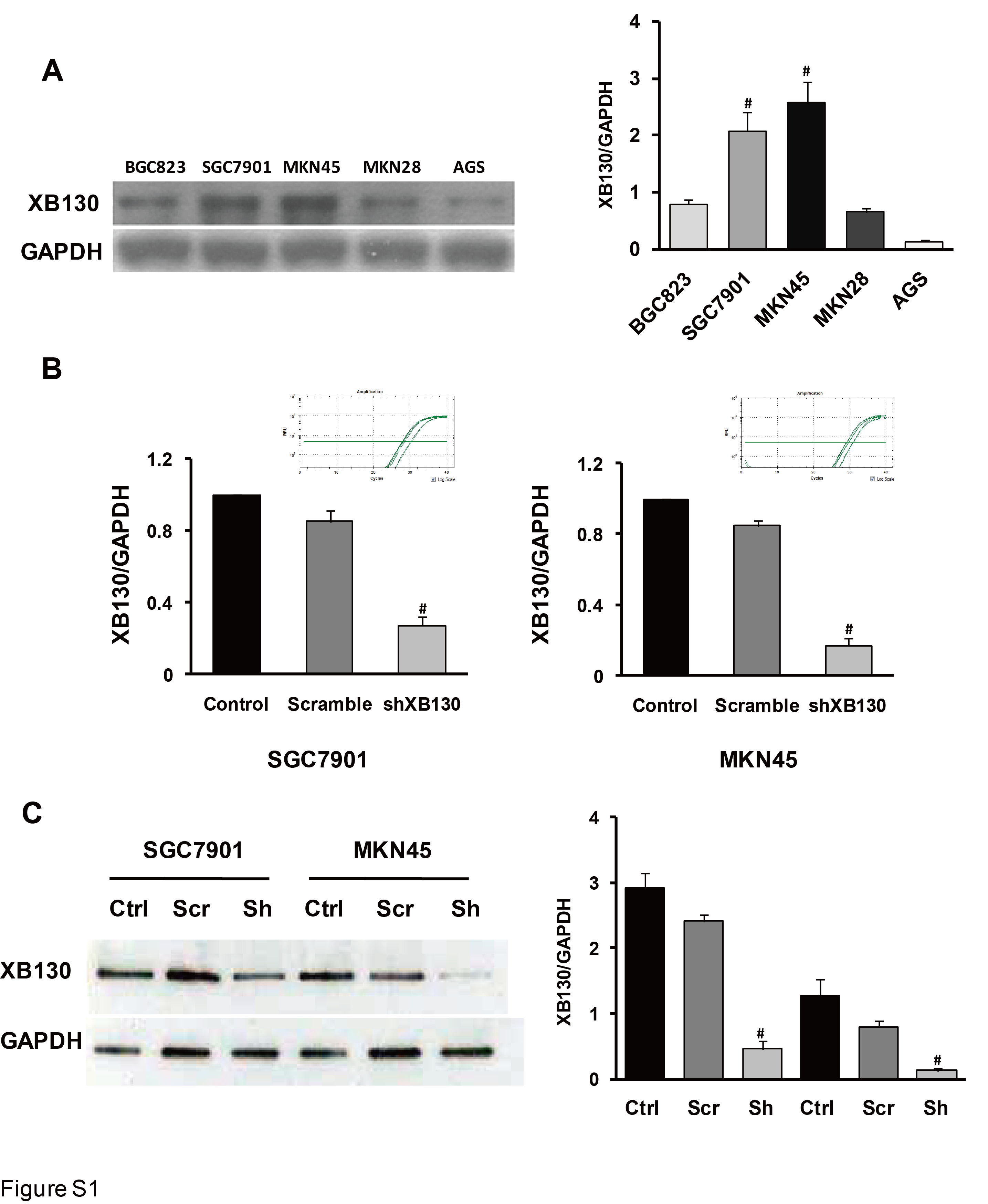

Supplement: Additional file 1: Figure S1 — Baseline expression and silencing effect of XB130 in gastric cancer (GC) cell lines. (A) Baseline expression of XB130 in various GC cell lines. Knocking down effect of sh-XB130 in SGC7901 and MKN45 cell lines was evaluated. Real-time PCR (B) and Western blot (C) revealed that sh-XB130 effectively suppressed the expression of XB130 in both cell lines. [file 1479-5876-12-1-S1.tiff]

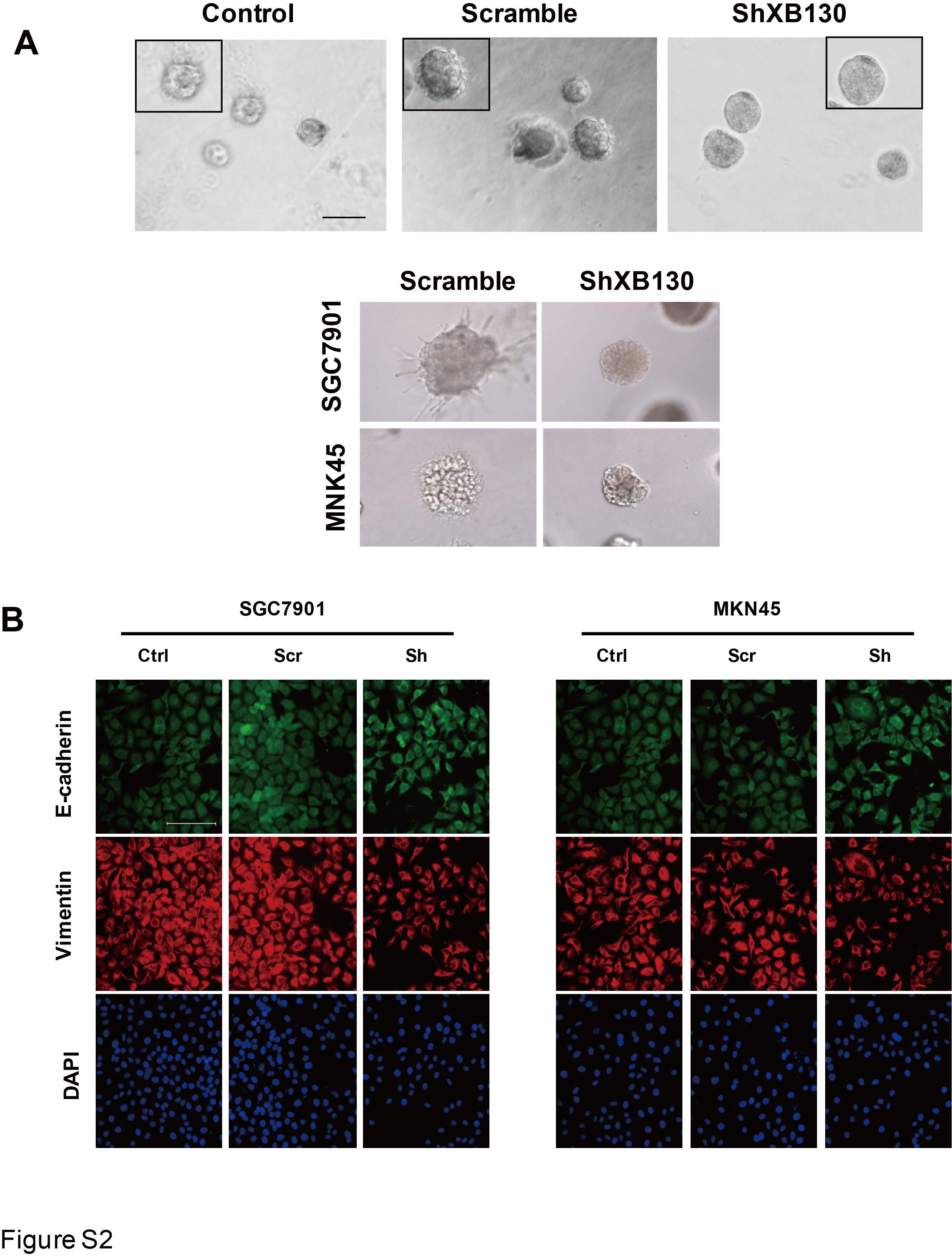

Supplement: Additional file 2: Figure S2 — Silencing XB130 changed the morphology of gastric cancer cells and altered the EMT-like associated proteins by cultured cells. (A) Matrigel 3D culture was performed to assess cell morphology. Aggressive protrusion-positive structures were found in the Scramble and Control group. In contrast, the invasiveness of sh-XB130 transfected cells was much lower and the cells were round spheroids with no or few protrusions. Pictures in upper line showed the view of 2–3 cells in each group under 100× magnification and the others were graphed under 200× magnification. (500 cells were incubated in each well). (B) E-cadherin and vimentin immunofluorescence staining. E-cadherin was significantly upregulated and vimentin was downregulated by XB130 knockdown. Scale bar = 100 μm. Experiments were repeated 3 times. [file 1479-5876-12-1-S2.tiff]
